# Supplementary figures and images for: Biotin-streptavidin-guided two-step pretargeting approach using PLGA for molecular ultrasound imaging and chemotherapy for ovarian cancer
Source: PeerJ. 2021 May 25;9:e11486. doi: 10.7717/peerj.11486 (PMC8162236; doi:10.7717/peerj.11486)

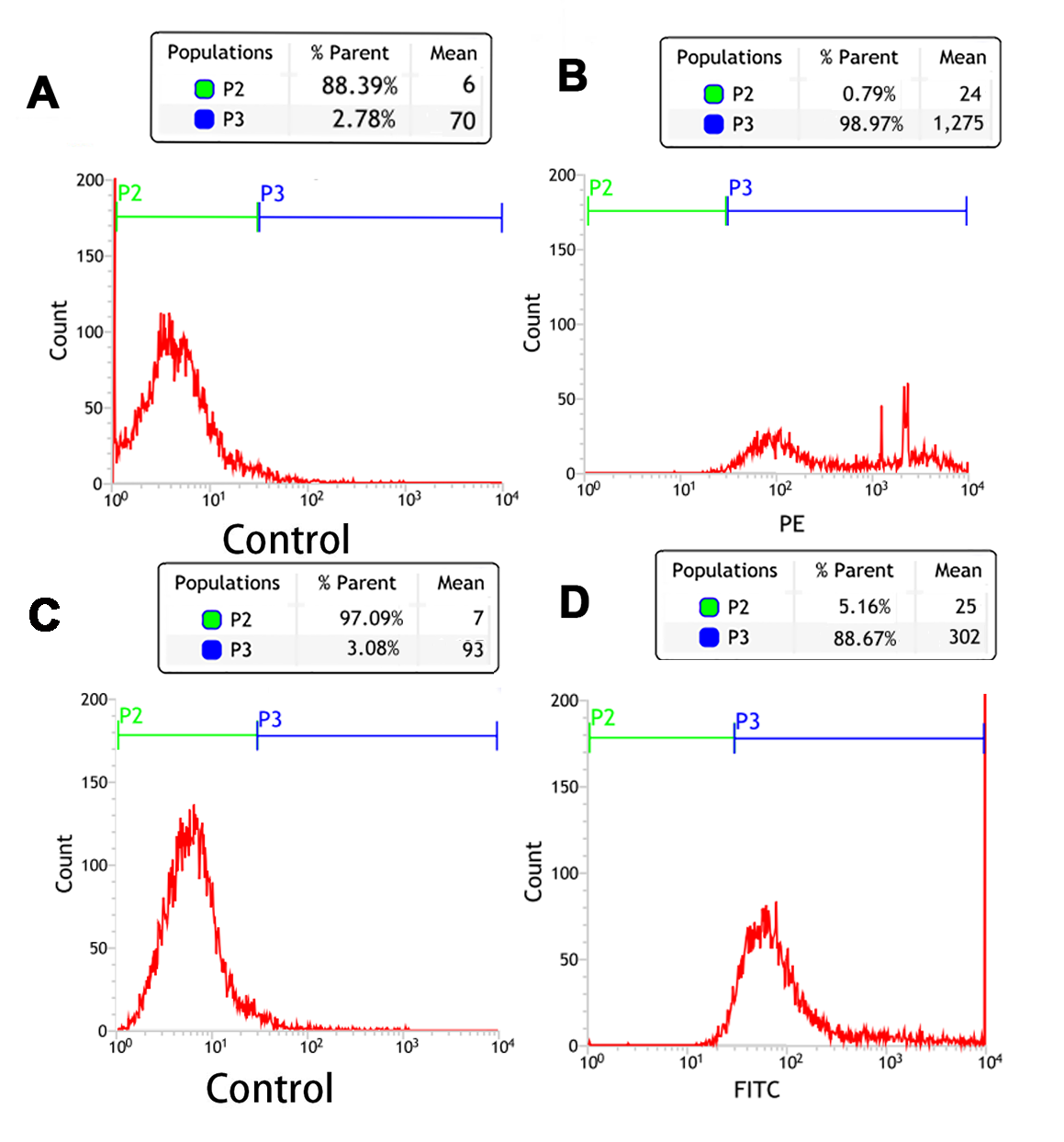

Supplement: Supplemental Information 2 — (A) Control; (B) SA group; (C) Control; (D) Ab group. [file peerj-09-11486-s002.png]
